# Outcomes after multiligament knee injury worsen over time: A systematic review and meta‐analysis

**DOI:** 10.1002/ksa.12442

**Published:** 2024-08-28

**Authors:** Antonio Klasan, Anne Maerz, Sven E. Putnis, Justin J. Ernat, Edouard Ollier, Thomas Neri

**Affiliations:** ^1^ AUVA UKH Steiermark Graz Austria; ^2^ Johannes Kepler University Linz Linz Austria; ^3^ Bristol Royal Infirmary University Hospitals Bristol and Weston NHS Foundation Trust Bristol UK; ^4^ Department of Orthopedic Surgery University of Utah Health Salt Lake City Utah USA; ^5^ University Hospital of Saint‐Etienne Saint‐Priest‐en‐Jarez France; ^6^ Laboratory of Human Movement Biology (LIBM EA 7424) University of Lyon ‐ Jean Monnet Saint‐Étienne France

**Keywords:** knee dislocation, meta‐analysis, multiligament knee injury, outcomes, posterior cruciate ligament based injury

## Abstract

**Purpose:**

Multiligament knee injuries (MLKIs) are devastating injuries that can have life‐long consequences. A management plan requires the decision to perform surgery or not, timing of surgery, consideration of repair versus reconstruction, reconstruction technique and reconstruction graft choice. The purpose of this study was to analyze development of clinical outcomes of MLKIs over time at a minimum of 2 years of follow‐up.

**Methods:**

Four databases were queried for surgical outcome‐based studies of MLKIs published from 01/2000 through 09/2022 with a minimum 2‐year follow‐up. Technique articles, nonoperative treatment, arthroplasty, pediatric and review articles were excluded. Study characteristics including design, number of patients, age, follow‐up period, anatomical region and posterior‐cruciate ligament (PCL)‐based injury were collected. Primary outcomes were Lysholm, International Knee Documentation Committee (IKDC) outcome scores and Tegner activity score. Random‐effects model analysis was performed.

**Results:**

After the application of inclusion and exclusion criteria, 3571 patients in 79 studies were included in the analysis. The mean age at surgery was 35.6 years. The mean follow‐up was 4.06 years (range 2–12.7). The mean Lysholm score at 2‐year follow‐up was 86.09 [95% confidence interval [CI]: 82.90–89.28], with a yearly decrease of −0.80 [95% CI: −1.47 −0.13], (*p* = 0.0199). The mean IKDC at 2 years was 81.35 [95% CI: 76.56–86.14], with a yearly decrease of −1.99 [95% CI: −3.14 −0.84] (*p* < 0.001). Non‐PCL‐based injuries had a higher IKDC 83.69 [75.55–91.82] vs. 75.00 [70.75–79.26] (*p* = 0.03) and Lysholm score 90.84 [87.10–94.58] versus 84.35 [82.18–86.52] (*p* < 0.01) than PCL‐based injuries, respectively.

**Conclusion:**

According to the present systematic review and meta‐analysis of MLKIs with minimum 2‐year follow‐ups, the patients who suffered an MLKI can expect to retain around 80‐85% of knee function at 2 years and can expect a yearly deterioration of knee function, depending on the score used. Inferior outcomes can be expected for PCL‐based injuries at 2 years postoperative.

**Level of Evidence:**

Level IV meta‐analysis.

**Abbreviations**
ACL: anterior cruciate ligament
IKDC: international knee documentation committee
KD: knee dislocation
LCL: lateral collateral ligament
MCL: medial collateral ligament
MLKI: multiligament knee injury
PCL: posterior cruciate ligament
PCLb: posterior cruciate ligament based

## INTRODUCTION

Multiligament knee injuries (MLKIs) and knee dislocation (KD) are rare but debilitating events for a patient [14]. The injury is defined as a disruption of at least two out of four major knee ligaments [82]. The incidence ranges between 0.02% and 0.2% of all orthopaedic injuries [70]. The distinction between the KD and MLKI is most commonly defined as a documented versus undocumented knee dislocation, with the former typically being associated with a higher incidence of nerve and vascular injury.

With improvements in MRI diagnosis, surgical approach and rehabilitation, outcomes have improved over the last 30–40 years [72, 84].

These injuries can be potentially life/limb threatening, typically require subspecialized treatment and are prone to postoperative complications [51], with long, often complex surgery and rehabilitation [66]. Due to this long recovery period, the success of the management is not typically determined until after over a year or more postoperatively [13].

The heterogeneity of the injury patterns, combined with the surgical timing options (acute, semi‐acute, delayed; single vs. multiple stages), surgical approach (repair and reconstruct), graft choice (autograft, allograft or synthetics) and fixation, creates a major issue in trying to perform a controlled study for better understanding of each of these factors. Additionally, posterior cruciate‐based (PCL‐based) injuries have worse clinical outcomes than non‐PCL‐based injuries [24, 27, 61, 76]. Finally, there is the issue of the potential of neurovascular injury, which can be a major risk factor for significantly worse outcomes [24, 27].

Previous systematic reviews have assessed timing of surgery [34, 64, 75, 92], outcomes depending on the mechanism of injury [8], return to work and sports [13] and compared repair and reconstruction [18, 92]. Presently, no meta‐analysis has tried to provide an answer to the question of long‐term functional outcomes after MLKIs and their development.

The primary purpose of the present systematic review and meta‐analysis was to analyze long‐term clinical and functional outcomes after MLKIs and their development over time, starting at minimum 2 years. The secondary purpose was to determine a potential difference between PCL‐based and non‐PCL based injuries. We hypothesized worsening of outcomes over time and worse outcomes for PCL‐based injuries.

## METHODS

The Preferred Reporting Items for Systematic Reviews and Meta‐Analyses extension statement for reporting of systematic reviews was followed. The PRISMA checklist is presented in Supporting Information: Appendix. This systematic review was registered with PROSPERO, the international prospective register of systematic reviews of the National Institute for Health Research, in April 2022 (registration number CRD42022364292).

### Search strategy

An independent review of citations in Medline, Embase, Web of Science and Cochrane Library after 1 January 2000 to 30 September 2022 was conducted independently by three authors (A.K., A.M. and S.P.). The search terms were as follows: ((multiligament) AND (knee)) AND (injury); (knee) AND (dislocation); (anterior cruciate ligament) AND (posterior cruciate ligament); (anterior cruciate ligament) AND (medial collateral ligament); (anterior cruciate ligament) AND (lateral collateral ligament); (anterior cruciate ligament) AND (posterolateral corner); (anterior cruciate ligament) AND (posteromedial corner); (posterior cruciate ligament) AND (posteromedial corner); (posterior cruciate ligament) AND (posterolateral corner); (posterior cruciate ligament) AND (medial collateral ligament); (posterior cruciate ligament) AND (lateral collateral ligament).

### Study selection

After exclusion of duplicates using Zotero® (Corporation for Digital Scholarship), all abstracts were screened by the three authors. Any disagreements among the authors about a study's potential inclusion were resolved by two other authors (J.E. and T.N.). References of included studies were additionally screened for further studies. We included clinical evidence level 4 and above and studies in skeletally mature adults, with minimum 2‐year clinical outcomes reported using either Lysholm [62] or IKDC [39] questionnaires. We excluded studies with concomitant osteotomies, partial or total knee replacements, fracture dislocations and studies with knee dislocations in a setting of congenital disorders. Excluded were non‐English studies, review articles, meta‐analyses, abstracts, case reports, biomechanical studies, cadaveric and animal studies and surgical techniques.

### Data extraction

Data extraction was performed by the same three authors and included study design, number of patients per group and patient characteristics. Mean follow‐up per group was used for the analysis. The data, however, are presented with the age and follow‐up averaged out across the cohort. Clinical outcomes extracted included Lysholm, IKDC and Tegner scores at the reported follow‐up. If the mean for any of the continuous variables was not reported in the study, it was calculated from the median, minimum and maximum using the estimate method described by Wan et al. [94]. Due to the heterogeneity of involved ligaments involved across studies and a current lack of a ligament‐oriented classification which predicts clinical outcomes, a subanalysis of outcomes was performed using the differentiation of studies explicitly including PCL‐based (PCLb) only, non‐PCL‐based (nPCLb) only or mixed (all ligament combinations, ALL) cohorts [6, 24, 27, 61, 76], where both PCL and non‐PCL‐based injuries in any possible combination of two or more ligaments were included.

### Risk‐of‐bias assessment

The methodological quality of each study was assessed independently by 4 review authors (A.K., A.M., S.P. and T.N.) according to the Newcastle–Ottawa scale [95] and reported in Table 1.

**Table 1:** Included studies, by study design, quality, patients per group, age and follow‐up.

Author, year	Study design	Newcastle–Ottawa	Patients per group				Mean age	Mean follow‐up, years
Ishibashi 2020 [40]	Cohort study	Good	12		19		48.0	4.9
Khakha 2016 [44]	Case series	Poor	36				36.5	10.1
Tapasvi 2022 [89]	Case series	Poor	34				30.6	4.2
Jakobsen 2010 [41]	Case Series	Poor	27				31.5	3.8
Kim 2012 [47]	Cohort study	Poor	20		25		n.r.	2
Van der Wal 2016 [93]	Cohort study	Poor	16				35.5	5
Fanelli 2002 [14]	Case series	Poor	35				n.r.	6
Hua 2016 [36]	Case series	Poor	18				38.8	4.8
Kim 2011 [48]	Cohort study	Good	21		25		35.5	2
Kim 2013 [49]	Cohort study	Good	22		24		39.7	2.9
Helito 2021 [32]	Cohort study	Good	18	24		24	27.0	5.2
Strobel 2006 [87]	Case series	Poor	17				30.7	3.4
Denti 2015 [10]	Cohort study	Good	10		10		34.5	10.2
Zorzi 2013 [107]	Case series	Poor	19				29.0	3.2
Fanelli 2004 [15]	Case series	Poor	41				n.r.	3.2
Lee 2011 [55]	Case series	Poor	70				31.2	3.3
Lutz 2021 [61]	Cohort study	Good	11		21		32.0	4.8
Lee 2010 [56]	Cohort study	Good	28		16		31.8	4.1
Khanduja 2006 [46]	Case series	Poor	19				29.6	5.6
Bonadio 2017 [5]	Case series	Poor	13				32.0	3.7
Helito 2022 [31]	Cohort study	Poor	37		41		30.4	3.4
Levy 2015 [57]	Cohort study	Poor	61		64		33.8	5
Gauffin 2013 [21]	Case series	Poor	4				n.r.	8
Zaffagnini 2011 [102]	Cohort study	Good	32		19		36.0	3.3
LaPrade 2018 [54]	Cohort study	Good	31		69		33.5	2.9
Millett 2004 [67]	Case series	Poor	19				35.7	3.8
Ranger 2011 [78]	Case series	Poor	71				38.5	4.5
Dekker 2021 [9]	Cohort study	Poor	50		19		38	3.6
Helito 2014 [30]	Case series	Poor	9				29.9	2.3
Moatshe 2017 [68]	Case series	Poor	65				36.0	12.7
Shelbourne 2007 [83]	Case series	Poor	21				21.4	4.6
Fanelli 2014 [17]	Cohort study	Poor	9	22		13	31.0	10.0
Djebara 2022 [11]	Case series	Poor	29				30.2	7.5
Plancher 2008 [77]	Cohort study	Good	31		19		26.0	8.3
Hirschmann 2010 [33]	Cohort study	Good	31	20		23	30.3	12.0
Zhang 2022 [105]	Cohort study	Poor	11		9		30.9	13.1
Li 2019 [59]	Case series	Poor	49				32.0	2.6
Godin 2017 [23]	Case series	Poor	20				17.7	3.1
Billières 2020[3]	Case series	Poor	20				28.3	2.5
Westermann 2019 [97]	Cohort study	Good	19		15		27.2	6
Freychet 2020 [20]	Cohort study	Good	20		20		29.5	4.8
Zhang 2021 [104]	Cohort study	Good	57		31		32.4	3.8
Hongwu 2018 [35]	Case series	Poor	13				37.8	2.7
Engebretsen 2009 [12]	Case series	Poor	85				31.0	6
Görmeli 2015 [25]	Cohort study	Poor	9		12		31.1	3.4
Mygind‐Klavsen 2017 [71]	Cohort study	Poor	77		119		34	5.9
Hanley 2017 [28]	Cohort study	Poor	25		9		25.7	6
Woodmass 2018 [98]	Cohort study	Poor	31		31		33.5	5.6
Jung 2008 [43]	Cohort study	Good	19		20		33.5	2.9
Van Gennip 2020 [22]	Case series	Poor	11				30.5	2
Woodmass 2018 [100]	Case series	Poor	20				30.7	4.4
Yang 2013 [101]	Case series	Poor	60				37.8	3
Burton 2020 [7]	Cohort study	Good	23		11		37.2	6.5
Li 2021 [58]	Cohort study	Poor	61		34		42.8	2
Woodmass 2017 [99]	Case series	Poor	23				26.7	7.5
Sanders 2018 [81]	Case series	Poor	61				32.0	3.8
Barrett 2018 [2]	Case series	Poor	32				30.0	3.3
Mardani‐Kivi 2019 [63]	Case series	Poor	28				30.9	3.0
Lo 2009 [60]	Case series	Poor	11				33.0	4.6
Zhao 2006 [106]	Case series	Poor	12				27.0	2.7
Zhang 2014 [103]	Case series	Poor	21				39.6	3.3
Osti 2010 [74]	Case series	Poor	22				28.8	3.0
LaPrade 2019 [53]	Cohort study	Good	153		41		34.5	3.5
Khan 2022 [45]	Cohort study	Good	14		13		35.8	2
Harner 2004 [29]	Cohort study	Poor	19		12		28.4	3.6
Angelini 2015 [1]	Case series	Poor	14				29.3	4.1
Ibrahim 2008 [37] i	Case series	Poor	26				27.3	4.4
Ibrahim 2013 [38]	Case series	Poor	20				26.4	3.6
Bin 2007 [4]	Case series	Poor	15				30.4	7.4
King 2016 [50]	Cohort study	Good	24		32		34	6.4
Fanelli 2012 [16]	Case series	Poor	35				n.r.	3 (2‐10)
Tzurbakis 2006 [90]	Cohort study	Poor	12	11		25	28.6	4.3
Shirakura 2000 [85]	Cohort study	Good	14		11		32.2	5.9
Stannard 2005 [86]	Cohort study	Poor	35		22		33	2.8
Jokela 2021 [42]	Cohort study	Good	18		7		43.1	6.9
Sundararajan 2018 [88]	Cohort study	Poor	31		14		39	3
Werner 2013 [96]	Cohort study	Good	192		23		33.8	5.8
Mariani 2001 [65]	Case series	Poor	14				25.1	3
Richter 2002 [79]	Cohort study	Poor	49	14		26	33.5	8.2

### Data analysis

The summary statistics for Lysholm, IKDC and Tegner, both overall and in prespecified subgroups, were generated using a random‐effect model with the corresponding 95% confidence intervals (95% CIs). For each meta‐analysis, statistical heterogeneity among studies was explored using the *I* ^2^ statistic. The influence of heterogeneity on the variability of summary statistics was illustrated by computing the 95% prediction intervals (95% PIs) both overall and in prespecified subgroups. The correlation between follow‐up duration and clinical scores was investigated by performing a linear meta‐regression. A P‐value for the association <0.05 was considered statistically significant. Statistical analysis was performed using R 4.3.2 (R Foundation).

## RESULTS

### Study inclusion and characteristics

**Figure 1:**
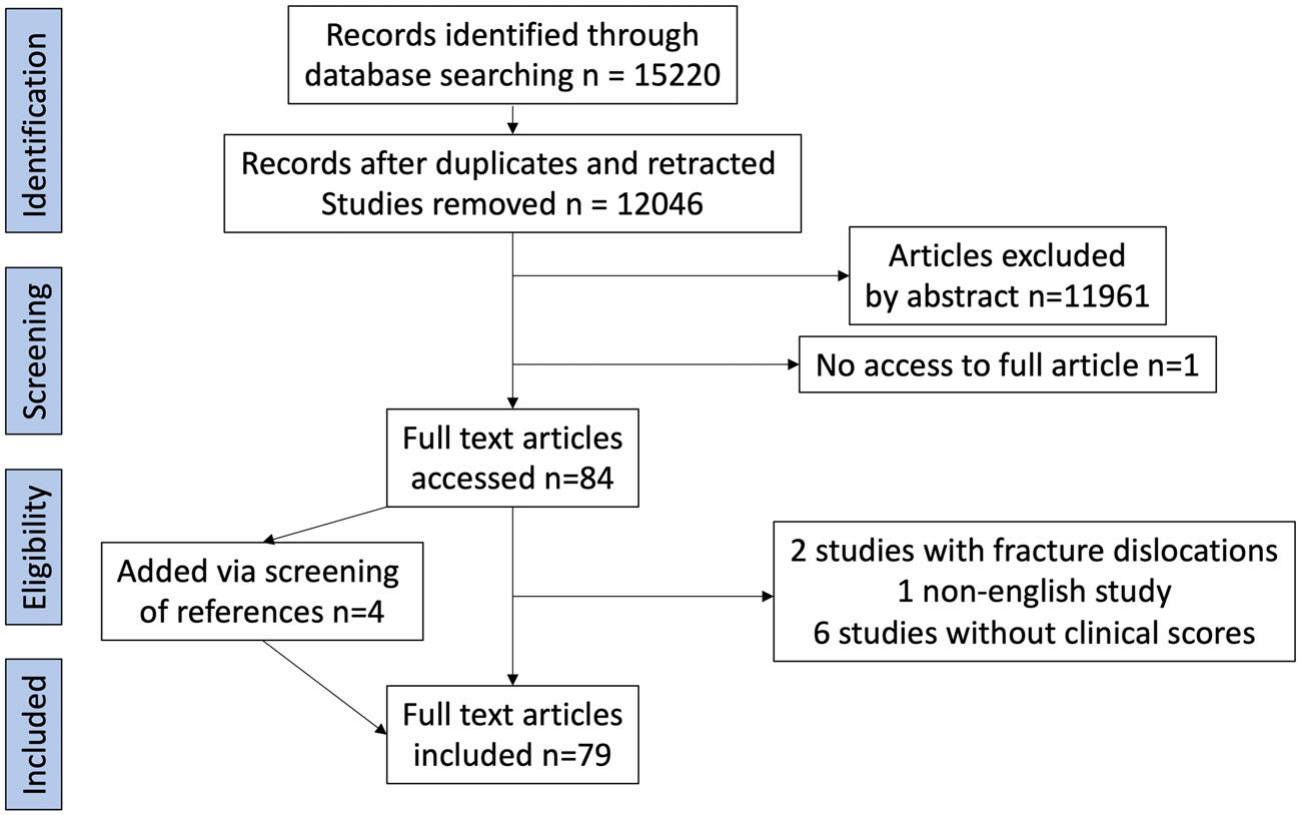
Flow chart of study inclusion.

After application of inclusion and exclusion criteria (Figure 1), 79 studies were included in the meta‐analysis, Table 1. All studies were either case series or cohort studies. A total of 3571 patients with MLKIs and KD were included in the meta‐analysis. The mean patient age was not reported in five studies [14, 15, 16, 21, 47], with the mean age at surgery across the studies of 35.6 years (range: 17.7–48.0 years). The mean follow‐up was 4.06 years (range: 2–12.7 years).

Studies were further divided into PCLb, nPCLb and ALL, Table 2. Management included non‐operative, primary surgical repair and surgical reconstruction with autograft, allograft, synthetic graft or a combination thereof, Table 2. The majority of studies included all injury patterns, Table 2.

**Table 2:** Included studies by PCL presence of PCL injury, surgical technique and anatomical region.

Author, year	PCL‐based	Surgical technique per group (repair vs. reconstruction vs. combination)			Anatomical region per group						
Ishibashi 2020 [40]	Yes	Repair	Combined		Combined	Combined					
Khakha 2016 [44]	Yes	Combined			Combined						
Tapasvi 2022 [89]	No	Reconstruction autograft			Combined						
Jakobsen 2010 [41]	Combined	Reconstruction autograft			LCL or PLC						
Kim 2012 [47]	Yes	Reconstruction allograft	Reconstruction allograft		Combined	Combined					
Van der Wal 2016 [93]	Combined	Reconstruction allograft			Combined						
Fanelli 2002 [14]	Yes	Combined			Combined						
Hua 2016 [36]	Yes	Repair			Combined						
Kim 2011 [48]	Yes	Combined	Combined		ACL + PCL + LCL/PLC		ACL + PCL + LCL/PLC				
Kim 2013 [49]	Yes	Reconstruction allograft	Reconstruction allograft		ACL + PCL + LCL/PLC		ACL + PCL + LCL/PLC				
Helito 2021 [32]	Combined	Reconstruction autograft	Reconstruction autograft		ACL + PCL + LCL/PLC	ACL + PCL + LCL/PLC				ACL + PCL + LCL/PLC	
Strobel 2006 [87]	Yes	Reconstruction autograft			ACL + PCL + LCL/PLC						
Denti 2015 [10]	Yes	Reconstruction allograft	Reconstruction autograft		Combined	Combined					
Zorzi 2013 [107]	Yes	Reconstruction allograft			ACL + PCL + LCL/PLC						
Fanelli 2004 [15]	Yes	Combined			ACL + PCL + LCL/PLC						
Lee 2011 [55]	Yes	Combined			Combined						
Lutz 2021 [61]	Combined	Reconstruction autograft	Reconstruction autograft		ACL + PCL + LCL/PLC	ACL + PCL + LCL/PLC					
Lee 2010 [56]	No	Reconstruction autograft	Reconstruction autograft		ACL + PCL + LCL/PLC	ACL + PCL + LCL/PLC					
Khanduja 2006 [46]	Yes	Combined			ACL + PCL + LCL/PLC						
Bonadio 2017 [5]	Yes	Reconstruction allograft			ACL + PCL + MCL						
Helito 2022 [31]	Combined	Combined	Combined		Combined	Combined					
Levy 2015 [57]	Combined	Combined	Combined		Combined	Combined					
Gauffin 2013 [21]	Yes	Combined			ACL + PCL + LCL/PLC						
Zaffagnini 2011 [102]	No	Reconstruction autograft	Reconstruction autograft		only ACL	ACL + PCL + MCL					
LaPrade 2018 [54]	Yes	Reconstruction allograft	Reconstruction allograft		only PCL	Combined					
Millett 2004 [67]	No	Combined			ACL + PCL + MCL						
Ranger 2011 [78]	Combined	Combined			Combined						
Dekker 2021 [9]	No	Reconstruction autograft	Reconstruction allograft		ACL + PCL + LCL/PLC	ACL + PCL + LCL/PLC					
Helito 2014 [30]	No	Combined			ACL + PCL + LCL/PLC						
Moatshe 2017 [68]	Combined	Combined			Combined						
Shelbourne 2007 [83]	Combined	Combined			Combined						
Fanelli 2014 [17]	Yes	Combined	Combined	Combined	Combined	Combined					Combined
Djebara 2022 [11]	Combined	Reconstruction autograft			Combined						
Plancher 2008 [77]	Combined	Combined	Non‐operative		Combined	Combined					
Hirschmann 2010 [33]	Yes	Combined	Combined		Combined	ACL + PCL + MCL	ACL + PCL + LCL/PLC				Combined
Zhang 2022 [105]	Combined	Combined	Non‐operative		Combined	Combined					
Li 2019 [59]	Yes	Reconstruction allograft			ACL + PCL + LCL/PLC						
Godin 2017 [23]	Combined	Reconstruction allo‐ or autograft			Combined						
Billières 2020[3]	Combined	Reconstruction allograft			Combined						
Westermann 2019 [97]	No	Combined	Repair		ACL + PCL	ACL + PCL					
Freychet 2020 [20]	Combined	Combined	Combined		Combined	Combined					
Zhang 2021 [104]	Yes	Combined	Combined		Combined	Combined					
Hongwu 2018 [35]	Yes	Combined			Combined						
Engebretsen 2009 [12]	Yes	Combined			Combined						
Görmeli 2015 [25]	Combined	Reconstruction allograft	Combined		PLC	Combined					
Mygind‐Klavsen 2017 [71]	Yes	Combined	Combined		only PCL	Combined					
Hanley 2017 [28]	Combined	Repair	Reconstruction allograft		Combined	Combined					
Woodmass 2018 [98]	Combined	Combined	Combined		Combined	Combined					
Jung 2008 [43]	Yes	Combined	Combined		ACL + PCL + LCL/PLC	ACL + PCL + LCL/PLC					
Van Gennip 2020 [22]	Combined	Reconstruction allograft			Combined						
Woodmass 2018 [100]	Combined	Combined			Combined						
Yang 2013 [101]	Combined	Reconstruction allograft			Combined						
Burton 2020 [7]	Combined	Repair	Reconstruction autograft		Combined	Combined					
Li 2021 [58]	Combined	Combined	Combined		Combined	Combined					
Woodmass 2017 [99]	Combined	Combined			Combined						
Sanders 2018 [81]	Combined	Combined			Combined						
Barrett 2018 [2]	Combined	Reconstruction allograft			Combined						
Mardani‐Kivi 2019 [63]	Yes	Reconstruction allograft			ACL + PCL + MCL + LCL						
Lo 2009 [60]	Yes	Reconstruction autograft			Combined						
Zhao 2006 [106]	Yes	Reconstruction autograft			Combined						
Zhang 2014 [103]	No	Reconstruction allograft			ACL + PCL + MCL						
Osti 2010 [74]	No	Combined			ACL + PCL + MCL						
LaPrade 2019 [53]	Combined	Combined	Combined		Combined	Combined					
Khan 2022 [45]	Combined	Combined	Combined		Combined	Combined					
Harner 2004 [29]	Yes	Combined	Combined		Combined	Combined					
Angelini 2015 [1]	Yes	Reconstruction allograft			Combined						
Ibrahim 2008 [37] i	Yes	Combined			Combined						
Ibrahim 2013 [38]	Yes	Reconstruction autograft			ACL + PCL + LCL/PLC						
Bin 2007 [4]	Yes	Combined			Combined						
King 2016 [50]	Yes	Combined	Combined		ACL + PCL + MCL	ACL + PCL + LCL/PLC					
Fanelli 2012 [16]	Yes	Combined			Combined						
Tzurbakis 2006 [90]	Combined	Combined	Combined		ACL	ACL + PCL + LCL/PLC		ACL + PCL + MCL or ACL + PCL + LCL/PLC			
Shirakura 2000 [85]	No	Repair	Non‐operative		ACL + PCL + MCL	ACL + PCL + MCL					
Stannard 2005 [86]	Combined	Repair	Reconstruction allograft		Combined	Combined					
Jokela 2021 [42]	Yes	Combined	Combined		ACL + PCL + MCL prox	ACL + PCL + MCL dist					
Sundararajan 2018 [88]	Yes	Combined	Combined		ACL + PCL + MCL	ACL + PCL + LCL/PLC					
Werner 2013 [96]	Combined	Combined	Combined		Combined	Combined					
Mariani 2001 [65]	Yes	Reconstruction autograft			Combined						
Richter 2002 [79]	Yes	Repair	Reconstruction allo‐ or autograft	Non‐operative	Combined	Combined			Combined		

Abbreviations: ACL, anterior cruciate ligament; LCL, lateral collateral ligament; MCL, medial collateral ligament; PCL, posterior cruciate ligament.

### Clinical outcomes

**Figure 2:**
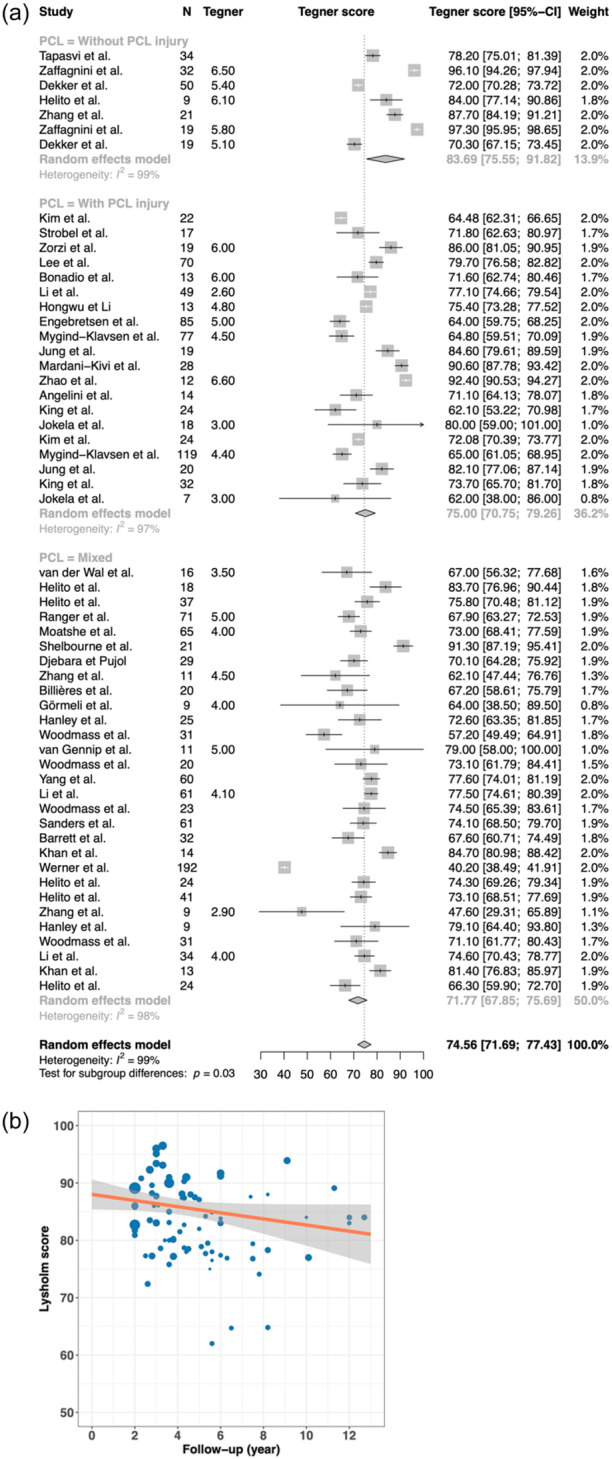
(a) Forest plot of outcomes according to the Lysholm score. Reported are authors, number of patients, mean Lysholm score, forrest plot and weight. (b) Graph demonstrating the development of Lysholm score over time. Each dot represents a study, the size of the dot represents the size of the cohort (weight), x‐axis is the follow‐up and y‐axis is the Lysholm score. The orange line represents the development of the mean score over time. CI, confidence interval.

Mean Lysholm score at 2 years follow‐up is 86.09 [95% CI: 82.90–89.28], as a starting point for patient‐reported outcomes in the present meta‐analysis, Figure 2a. The yearly decrease of Lysholm score was −0.80 [95% CI −1.47 −0.13], (*p* = 0.0199), Figure 2b. Some long‐term follow‐up outliers existed, but the overall trend toward worsening of outcomes over time is visible.

**Figure (3):**
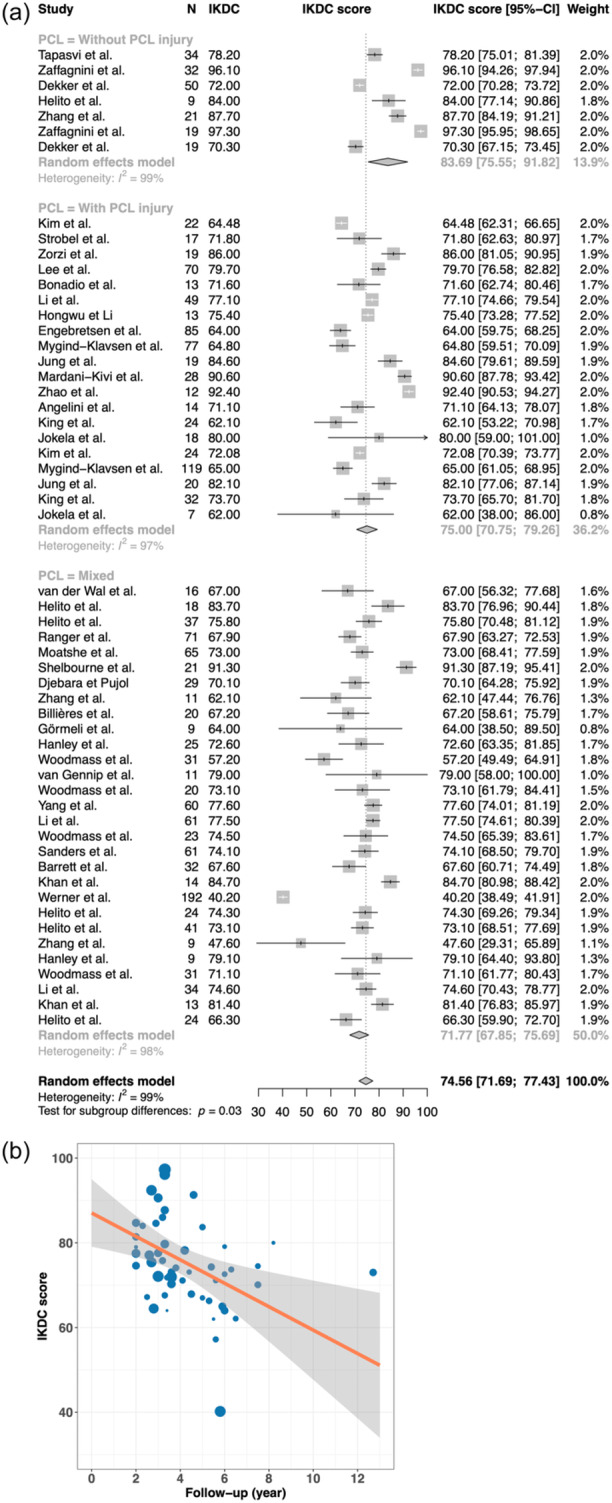
(a) Forest plot of outcomes according to the IKDC score. Reported are authors, number of patients, mean IKDC score, forest plot and weight. (b) Graph demonstrating the development of IKDC score over time. Each dot represents a study, the size of the dot represents the size of the cohort (weight), x‐axis is the follow‐up and y‐axis is the IKDC score. The orange line represents the development of the mean score over time. CI, confidence interval; IKDC, International Knee Documentation Committee.

In studies where IKDC was used for evaluation, at 2 years, the mean score was 81.35 [95% CI 76.56–86.14], a lower starting point than when Lysholm was used. There was a higher yearly decrease of −1.99 [95% CI −3.14 −0.84] (*p* < 0.001) (Figure 3a,b).

The mean Tegner activity scale at 2 years was 5.0527 [95% CI 4.5312–5.5742]. Per year, there was a decrease of −0.0840 [95% CI −0.1796; 0.0116] (*p* = 0.085).

**Figure 4:**
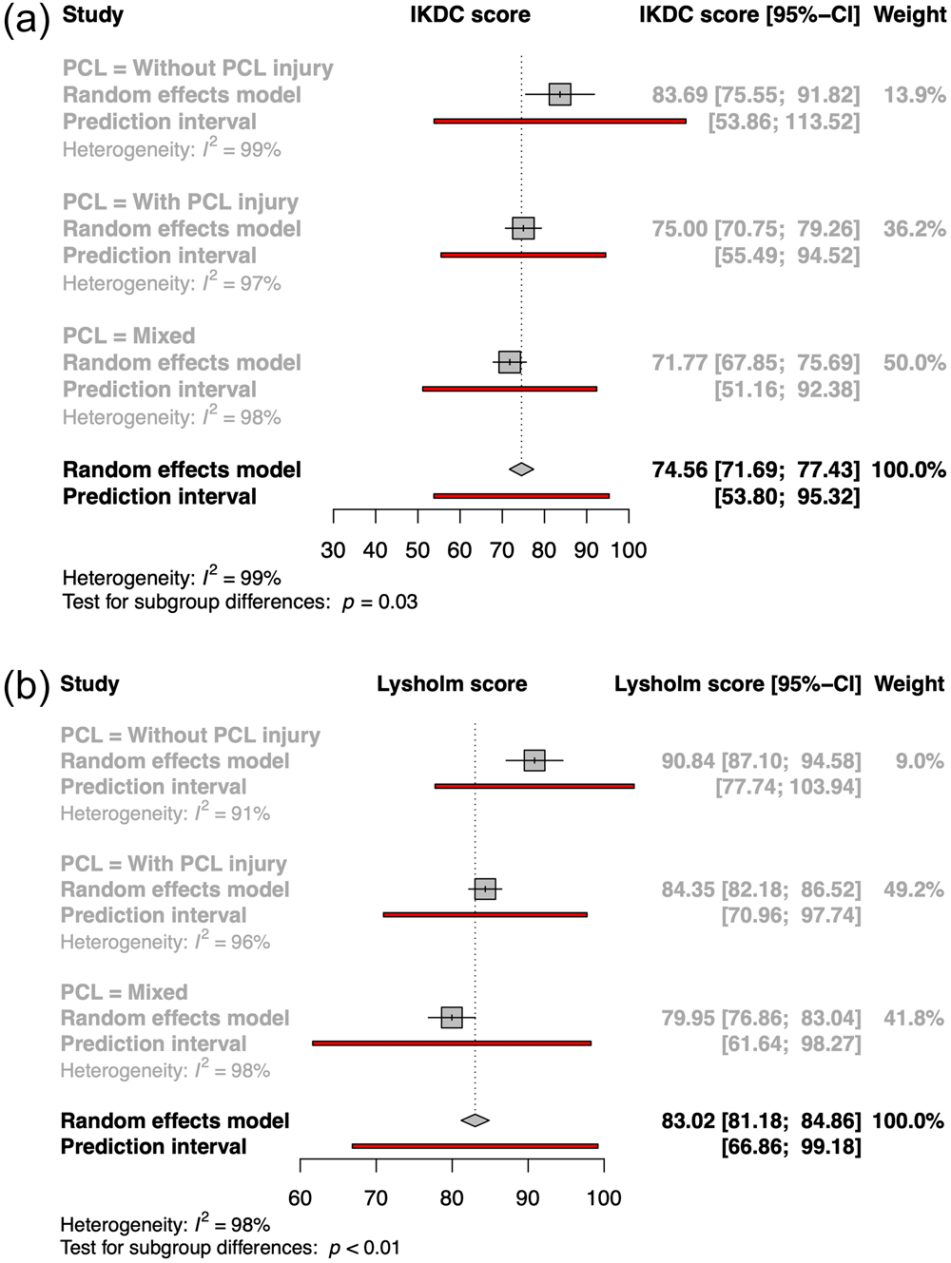
(a) Forest plot of outcomes based on the presence of a PCL injury according to the Lysholm score. (b) Forest plot of outcomes based on the presence of a PCL injury according to the IKDC score. CI, confidence interval; IKDC, International Knee Documentation Committee; PCL, posterior cruciate ligament.

Although the majority of studies investigated a mixed cohort, for both Lysholm and IKDC, there was a difference of mean values between PCL‐based and non‐PCL‐based injuries, Figure 4a,b, for the same mean follow‐up time. Lysholm score at 2 years uncontrolled for PCL‐based injuries was 86.09, compared to 90.84 without a PCL injury and 84.35 with PCL‐based injury. Similarily, IKDC score for non PCL‐based injuries was 83.69 compared with 75.00 for PCL‐based injuries.

## DISCUSSION

The most important finding of the present systematic review and meta‐analysis is the worsening of the clinical outcomes over time, when measured with Lysholm, IKDC and Tegner scores. Furthermore, long‐term results of PCLb injuries are significantly worse than those in nPCLb injuries at minimum 2‐year follow‐ups and with progression over time.

In the present systematic review, 6 studies investigated ≥10 year follow‐up [10, 17, 33, 44, 68, 105]. On average, the present meta‐analysis estimates the Lysholm score at 10 years to around 78, providing a good overall estimate and overlapping with the results of these long‐term studies, but this is very dependent on the type of injury. If this is compared to results at 2 years, the difference is clear. In the lack of similar studies on multiligament injuries, a comparison to ACL reconstruction and long‐term outcomes can be performed. After 20 years, the results of ACL reconstruction are considered satisfactory but with 10% of patients having residual laxity [26]. Osteoarthritis prevalence is high, especially in patients with concomitant injuries to the cartilage, meniscus [52] and extensor mechanism [69], as well as delayed surgery [26]. The heterogeneity of the possible injured‐ligament combinations renders any systematic review complex: surgery or no surgery, repair or reconstruct, what to use for reconstruction, which technique to use and, finally, when to do the surgery. Several systematic reviews on the subject of MLKIs have been published in the literature, addressing various aspects. None have provided an answer to long‐term recovery and progression of functional outcomes over time. It can be hypothesized that the injury pattern of MLKIs, including neurovascular damage, causes significantly faster deterioration of knee function at long‐term follow‐ups.

Previous systematic reviews in MLKIs mostly investigated differences in acute versus delayed surgical treatment. Marder et al. investigated acute versus delayed intervention in MLKI in 31 studies; however, they did not elucidate whether acute or delayed intervention produced superior outcomes [64]. Hohmann et al. [34] on the other hand, investigated eight studies and found early surgery to have better outcomes than delayed surgery. Interestingly, Özbek et al. [75] investigated 36 studies and found that early surgery in a setting of more than three ligaments increases the odds of stiffness (OR = 0.45). Vermeijden et al. [91] investigated both isolated ACL (16 studies) and MLKI (14 studies) and found no differences in early versus delayed surgery for both ACL and MLKI injuries. In the present study, with a larger number of included studies, this sub‐analysis has not been performed due to the heterogeneity of timelines between the studies. Given that there is conflicting literature reporting on the benefits/risk of early versus delayed surgery, we elected to study these outcomes collectively, with a larger goal focussed on projected outcomes. Although, admittedly, we understand that failure to control for staging/timing is a limitation of this meta‐analysis.

Dean et al. [8] performed a meta‐analysis comparing high‐ and low‐energy MLKI in 15 studies. The authors found improved Tegner scores for low‐energy injuries but no differences in Lysholm or IKDC score at 5.3 years postoperatively. The clinical outcomes reported in the study by Dean et al. are lower than the scores found in the present study, using the 2‐year follow‐up average score and the yearly loss of score, which might be due to the majority of studies including injuries of all four major ligaments. Everhart et al. [13] investigated return to work or sport after MLKI in 21 studies, finding a return to sport rate at 60% and most patients going back to work. However, most patients returned to work with frequent workplace or job duty modifications.

Fortier et al. [18] investigated injuries of the posterolateral corner only and found significantly higher success rate of reconstruction versus repair. The authors also conclude that the heterogeneity in the literature is present even if only one anatomical region/ligament is investigated. Vicenti et al. [92] tried to answer three questions in a systematic review: surgical repair or non‐operative treatment, repair versus reconstruction and early versus late surgery. The authors concluded that there is no discernable “best” treatment but that reconstruction seems to work better and that surgery should be done within 3 weeks when possible [92].

Instead, a distinction PCL versus non‐PCL based (PCLb versus nPCLb) injuries was performed, due to the previously reported significantly worse outcomes of PCLb injuries in some studies [61, 76]. Currently, no classification or clear distinction between PCL‐based and non‐PCL‐based injuries is reported in the literature. Instead, there are individual studies investigating this issue [57, 70]. This was added to the present study mainly to increase awareness of the vast difference in outcomes between these two, which will hopefully drive a distinction in reporting in the literature. Overall, the present study found worse outcomes for PCLb injuries. The observed difference in IKDC is clinically detectable [19], and the difference in Lysholm is somewhat below the minimally clinically important difference [73], although this also depends on the procedure performed [80]. The principal difference is the fact that a PCLb injury more often signifies a knee dislocation, either with or without radiologic evidence of dislocation, which is a more severe MLKI [24]. The underlying issue with classifying these injuries is the fact that KD classification is used for MLKIs, where not every MLKI is a KD [27].

## LIMITATIONS

The limitation of the present study is the heterogeneity of the severity of injury, management, surgical techniques, follow‐up periods and preoperative data. Early versus late, repair versus reconstruction, allograft versus autograft and PCL‐based versus non‐PCL based are all parameters that add a significant number of permutations, virtually impossible to control for. Despite that, by applying wide search criteria and including 79 MLKI studies with minimum 2‐year follow‐ups, a decrease in the skewness of the data of potential outliers in terms of results can be expected. Even PCL‐based injuries have not been completely reported in the includes studies, but the observed difference reported in some smaller clinical studies and in this meta‐analysis open an important aspect to further investigate and discuss. Six studies that did not utilize Lysholm or IKDC were excluded, representing less than 10% of the overall studies. It is unlikely that these studies would alter the results significantly. Finally, neurovascular complications were not assessed in the study, both due to the heterogeneity of the injuries and lack of reporting.

## CONCLUSION

According to the present systematic review and metanalysis of MLKI with minimum 2‐year follow‐ups, the patients who suffered a MLKI can expect to retain around 80‐85% of knee function at 2 years and can expect a yearly deterioration of knee function, depending on the score used. Inferior outcomes can be expected for PCL‐based injuries at 2 years postoperative.

## AUTHOR CONTRIBUTIONS

All the authors devised the study. Antonio Klasan, Anne Maerz and Sven Edward Putnis did the screening, data extraction and the review. Justin J. Ernat resolved discrepancies. Antonio Klasan wrote the first draft; Thomas Neri and Justin J. Ernat revised it. All authors have read and approved the manuscript.

## CONFLICT OF INTEREST STATEMENT

A.K. is an associate editor for the Journal of Knee Surgery and Editorial Board Member of Archives of Orthopaedic and Trauma Surgery and Knee Surgery, Sports Traumatology, Arthroscopy. He has been paid for presentations by Arthrex, Implantcast and FH Ortho. S.E.P. and T.N. are consultants for FH Ortho. J.J.E. is a consultant for DePuy Synthes.

## ETHICS STATEMENT

PROSPERO Registration number CRD42022364292. Systematic review requires no consent.

## Supporting information

Supplementary Information

## Data Availability

All studies included are openly available. Our analysis can be shared upon request.
